# Pediatric femoral shaft fractures: treatment strategies according to age - 13 years of experience in one medical center

**DOI:** 10.1186/1749-799X-8-23

**Published:** 2013-07-17

**Authors:** Yaron Sela, Oded Hershkovich, Nir Sher-Lurie, Amos Schindler, Uri Givon

**Affiliations:** 1 Department of Orthopedic Surgery, Chaim Sheba Medical Center, Sackler Faculty of Medicine, Tel Aviv University, Tel Aviv 6997801, Israel; 2 Department of Pediatric Orthopedics, Chaim Sheba Medical Center, Sackler Faculty of Medicine, Tel Aviv University, Tel Aviv 6997801, Israel

**Keywords:** Femoral shaft fracture, Spica, Traction, Casting, Flexible nails, Plating, Antegrade trochanteric nail, External fixation, Children

## Abstract

**Objective:**

The objective of this study was to analyze our experience in treating femoral shaft fractures with different strategies, focusing on the first year after injury when the choice of method would have the greatest impact.

**Methods:**

We reviewed the medical records of all children treated for femoral shaft fractures in our institution between 1997 and 2010. They were divided according to therapeutic approach: spica cast, skin traction, titanium elastic nail (TEN), external fixator, intramedullary medullary nail (IMN), and plating.

**Results:**

The 212 patients included 150 boys and 62 girls (M/F ratio 2.4:1, mean age 5 years, range 0–16). The postoperative radiographic results demonstrated solid union in all patients, with no malunions. Of the 151 children in the spica cast group, 10 required re-manipulation and casting due to loss of reduction with unaccepted angulation, 10 had contact dermatitis, and 2 had fever and pressure sores. All 21 elastic nail group children underwent re-operation to remove the hardware: 3 had soft tissue irritation at the insertion points, and 3 had leg length discrepancy (LLD). Of the 14 external fixation patients, 4 had LLD, 1 had a pin tract infection, and 1 had a fracture through a pinhole after a fall. There were no complications in the 12 IMN patients, the 3 plating patients, or the 11 skin traction patients. LLD rates in the spica group were 10.5% higher compared to those in the control group (other treatment modalities) (*P* = .03).

**Conclusions:**

TEN treatment was superior to spica casting for children who had reached an average age of 4 years.

## Introduction

Femoral shaft fractures, typically caused by blunt trauma, are the most common major pediatric injuries treated by the orthopedic surgeon. Seventy percent of femoral fractures involve the shaft [1]. Femoral shaft fractures reportedly occur at a rate of approximately 20/100,000 children in the USA [2], representing 1.6% of all fractures in the pediatric population. Insofar as no operative technique had yielded consistently better results than casting, nonsurgical treatment continues to be the preferred and most cost-effective form of management for the preschool-age child with an isolated femoral fracture. The accepted methods are a Pavlik harness and/or splints for newborns or young infants and an immediate or early spica cast for older infants, toddlers, and young children [3]. For the school-age child with an isolated fracture, the impact of prolonged immobility has prompted the use of surgical techniques that permit rapid mobilization. The preferred therapeutic approach is dictated by the patient's age, fracture characteristics, and characteristic physical activities. In general, early closed reduction and application of a spica cast is an accepted treatment for most diaphyseal femoral fractures for children who are 5 years of age or younger [4]. In skeletally mature adolescents, the use of an antegrade solid intramedullary trochanteric nail has become the standard of treatment. The results of a recent survey of the members of the Pediatric Orthopaedic Society of North America indicate that surgery is the preferred treatment for older children, particularly those with high-energy injuries [5-10].

The optimal mode of treatment among the wide variety of surgical and nonsurgical treatment options for children between 5 and 15 years of age continues to be controversial [11]. Unlike younger children, patients in this intermediate age group have a higher risk of shortening and malunion from early closed reduction and the use of a spica cast. Two or three weeks of traction prior to the application of the cast can maintain length until early healing has occurred. Notably, the older children who are managed with traction and a spica cast may miss several months of school until full union has been achieved, and so various operative strategies have been used for them with the aim of avoiding the adverse physical, social, psychological, and financial consequences associated with prolonged immobilization. Those methods include flexible intramedullary and antegrade solid nails, external fixation, plates, and screws. Each procedure carries the risk of certain complications, particularly pin track infection and refracture after external fixation removal or osteonecrosis after fixation with a solid antegrade intramedullary nail (IMN). External fixation, however, does allow for early discharge from hospital, is less cumbersome than the hip spica cast, and can be effective for controlling the fracture position, theoretically leading to reduced rates of malunion [12,13]. Over the past 12 years, fixation with flexible IMNs has become a popular method for stabilizing femoral fractures in school-age children in North America, even though there has been little evidence to support that preference [14,15]. We retrospectively analyzed our experience in treating femoral shaft fractures by means of different treatment strategies. We focused on the first year after injury, reasoning that it is the period when the choice of treatment method would have the greatest impact. Our main objective was to compare the results of conservative treatment by spica casting with those of other types of treatment for femoral fractures in children of different ages.

## Patients and methods

We retrieved the medical records of all the patients treated for femoral shaft fractures in the Pediatric Orthopedic Surgery Unit of the Sheba Medical Center (Tel Hashomer, Israel) between January 1997 and January 2010. All the files of the patients were reviewed with the approval of the Sheba Medical Center IRB.

All patients underwent periodic radiographic evaluation and physical examinations. The minimal follow-up period of the children enrolled in this study was 12 months. Twenty-two patients were lost to follow-up and their information was incomplete, and so we calculated their results based on the available information (e.g., we had the records of 19 out of the 21 children treated with a titanium elastic nail (TEN) for leg length discrepancy (LLD), and those records showed that 3 of these 19 (15.8%) had LLD).

The patients were grouped according to the type of treatment they underwent: spica cast, skin traction (seven newborns that were treated with Bryant's skin traction), elastic nail, external fixator, IMN, and plating. The children who underwent conservative treatment with spica casting comprised the study group, and the children who underwent all of the other therapeutic approaches served as the controls.

Only the data on children for whom there was information on all the selected parameters were used for the statistical analysis of our results. We had to control for the characteristics of age and gender as well as the patterns of injury and type of fracture (e.g., high energy, side, etc.) of both the study and control groups. We applied propensity score matching (PSM; Appendix) to compare our two groups with others [16,17] who used it in a similar context. For checking the credibility of PSM, we compared between the explanatory variables (i.e., sex, side of injury, type of fracture) after matching the two groups, and this led to the exclusion of 17 controls and one patient from the spica cast group.

## Results

A total of 212 patients were enrolled in this study (Table 1). They included 150 boys and 62 girls (2.4:1) whose mean age was 5 years (range 0–16 years). The etiology of the fractures was a high-energy trauma in 53 patients (25%) at a mean age of 8 years: 41 of them were sustained in motor vehicle accidents and 12 resulted from falling from height. The 163 patients (75%) who were treated at a mean age of 4 years had suffered from low-energy trauma. The spica cast-treated group consisted of 151 patients whose average age was 3.5 years (0.5–14). The skin traction-treated group consisted of 11 patients (included 7 newborns treated with Bryant's skin traction) whose average age was 2 months (0–1 year). There were 21 patients in the elastic nail-treated group whose average age was 9.5 years (6–13.5), and the 14 patients in the external fixator-treated group had an average age of 10.6 years (4–14.5). The IMN group had 12 patients (1 patient with bilateral femoral fractures) whose average age was 14.4 years (9.5–16), and there were 3 patients in the plating group whose average age was 10.5 years (7.5–13). All the reported femoral fractures had a radiographically confirmed solid union regardless of treatment method.

**Table 1:** Demographic characteristics according to treatment group

**Demographic characteristics**	**Plating**	**IMN**	**Skin traction**	**Ex-Fix**	**TEN**	**Spica**
Number of patients	3	12	11	14	21	151
Average age (years)	10.5	14.4	0.2	10.6	9.7	3.5
Sex (M/F) (% of patients)	67:33	67:33	64:36	62:38	52:48	75:25
Side (L/R) (% of patients)	0:100	55:45	55:45	57:43	62:38	54:46
Type of fracture (% of patients)						
Spiral	33.3	10	50	0	30	67
Oblique	33.3	0	10	0	0	6
Transverse	33.3	80	40	50	70	20
Comminuted	0	10	0	30	0	1
Open	0	0	0	20	0	0
Buckle	0	0	0	0	0	6
Mechanism of injury (% of patients)						
Fall from height	0	8	0	8	5	6
Motor vehicle accident	0	42	0	75	50	9
Fall with minor trauma	100	50	100	17	45	81
Skating/biking	0	0	0	0	0	1.5
Multitrauma injury	0	0	0	0	0	2.5

*TEN* titanium elastic nail, *Ex-Fix* external fixator, *IMN* intramedullary medullary nail.

The treatment outcome for each group is detailed in Table 2. There were no cases of malunion. The complications and side effects of each treatment arm were as follows. There were none in the 11 skin traction subjects. There were 33 reported complications in the 151 spica cast-treated children: these included 7 cases of LLD >2 cm of shortening and 4 cases of LLD >1 cm lengthening of the treated leg (yielding a 7.3% LLD rate), 10 cases of re-manipulation and casting due to loss of reduction with unaccepted angulations, 10 cases of contact dermatitis, and 2 cases of fever and pressure sores. The spica cast group had an overall union rate of 100%, a normal LLD rate of 92.7%, and an 85.4% rate of complication-free procedures (Table 2). After applying PSM, there were 10.5% more LLDs among the spica cast-treated children compared to all other treatment groups combined.

**Table 2:** Treatment outcome according to treatment group

**Outcome**	**Plating**	**IMN**	**Skin traction**	**Ex-Fix**	**TEN**	**Spica**
Union^a^	100	100	100	100	100	100
Nonunion	-	-	-	-	-	-
Malunion	-	-	-	-	-	-
LLD (% of patients)						
Normal LLD	100	63.6	100	67	84.2	92.7
LLD >2 cm of shortening	-	-	-	16	5.3	4.6
LLD >1 cm of lengthening	-	27.3	-	16	10.5	2.7
Total LLD	-	27.3	-	33	15.8	7.3
Complications (% of patients)						
None	100	100	100	75	91.5	85.4
Local infection	-	-	-	8.3	-	1.3
Limitation of range of motion	-	-	-	8.3	-	0.66
Avascular necrosis	-	-	-	-	-	-
Contact dermatitis	-	-	-	-	-	6.6
Re-manipulation and casting	-	-	-	-	-	6.6
Re-fracture	-	-	-	8.3	-	-
Neurovascular deficit	-	-	-	-	-	-
Soft tissue irritation at insertion site (for TEN procedures)	-	-	-	-	9.5	-
Change in type of treatment	-	-	-	-	-	0.66

^a^Radiographic confirmation of solid union regardless of treatment method. There were no cases of malunion. *TEN* titanium elastic nail, *Ex-Fix* external fixator, *IMN* intramedullary medullary nail, *LLD* leg length discrepancies.

The descriptive statistics (Table 3) revealed that 117 patients treated by the spica cast were significantly younger than the children in the control group (by about 5 years, *P* < .0001). They also differed significantly by the type of fracture (spiral and transverse; *P* < .001). Having found that the patient's age had a strong effect on the outcome, it was not sufficient to merely control for this parameter, and so we used PSM in order to compare patients similar in all the selected characteristics (age, fracture type, injury energy, and the side of injury) in both groups. Table 4 demonstrates that after sorting the differences between the explanatory variables (i.e., patient's characteristics), there was no longer any significant difference between those parameters, particularly the age of the patient. Analysis of the naïve data without applying PSM revealed that spica treatment had a successful outcome (i.e., result of equal leg length and no LLD at the end of the treatment) in 11.3 percentage points less compared with other treatments, but the difference did not reach a level of significance. When applying PSM, the outcome of the spica cast treatment significantly decreased the chance of healing with no LLD by 10.5% compared to the other examined treatment arms (*P* < .01) (Table 4).

**Table 3:** Descriptive statistics comparing spica cast treatment to the control group

**Treatment (yes/no spica cast)**^**a**^	**All patients**	**Control group**	**Spica casting**	**The disparity**	***P *value**
Frequency	150	33	117		
Result	0.76	0.85	0.74	*−0.113*	0.138
Male	0.70	0.64	0.72	0.082	0.393
Age	4.62	8.32	3.57	−4.749	*0.0001*
Left side	0.57	0.64	0.56	−0.081	0.407
Spiral fracture	0.60	0.33	0.68	0.342	*0.006*
Transverse fracture	0.29	0.61	0.21	−0.401	*0.000*
Oblique fracture	0.05	0.03	0.06	0.030	0.433
High energy	0.21	0.39	0.16	−0.232	*0.017*

The control group includes titanium elastic nail, external fixator, intramedullary medullary nail, and plating. ^a^Regression result is (−0.113, 0.03). The italicized entries signify the change in the groups' disparity before and after the PSM was applied on the date.

**Table 4:** The calculated statistics comparing spica cast treatment to the control group after applying PSM

**Treatment (yes/no spica cast)**^**a**^	**All patients**	**Control group**	**Spica casting**	**The disparity**	***P *value**
Frequency	132	16	116		
Result	0.75	0.88	0.73	*−0.142*	0.148
Male	0.71	0.69	0.72	0.028	0.828
Age	3.56	4.23	3.47	−0.751	0.5238
Left side	0.57	0.69	0.55	−0.136	0.303
Spiral fracture	0.67	0.69	0.67	−0.015	0.907
Transverse fracture	0.21	0.25	0.21	−0.043	0.719
Oblique fracture	0.06	0.06	0.06	−0.002	0.974
High energy	0.17	0.25	0.16	−0.086	0.471

The control group includes titanium elastic nail (15 cases) and external fixator (1 case). ^a^Regression result is (−0.105, <0.01). The italicized entry signifies the change in the groups' disparity before and after the PSM was applied on the date.

All 21 patients in the elastic nail-treated group underwent a second operative procedure 6–12 months later to remove the implant. There were three cases of soft tissue irritation at the insertion points and three cases of LLD (Table 2). The average age of the children in this group was 9.7 years. Over one half (55%) of the fractures were high-energy (55%) and 70% were transverse (Table 1).

Twelve of the 14 external fixator-treated children had sustained high-energy fractures with comminution or open fractures (Table 1). Their average age was 10.6 years. Two of them had LLD. One external fixator patient had pin tract infection which resolved after debridement and oral antibiotic treatment. One child needed knee joint manipulation due to restricted range of motion. One case of fracture through an external fixator pinhole occurred due to a second fall 2 months after the initial operation: he was treated successfully by extension of the external fixator over the new fracture (Table 2).

The average age of the 12 IMN-treated patients was 14.4 years: 80% had transverse fractures, of which one half were high-energy (Table 1). There were no cases of avascular necrosis of the femoral head or any other complications (Table 2).

There were only three children in the plating group. Their average age was 10.5 years, and there were no surgery-associated complications.

Our records indicated that the spica cast approach was by far our most frequently implemented type of treatment and that its relative use was stable throughout the 13-year study period. The TEN treatment gained in popularity in the later study years (2005–2009), and it was used from the age of 5 years up to the age of 16 years, most frequently for children in the 5- to 10-year age range.

Spiral and transverse fractures were the most common among the various types of fracture, and there were only a few comminuted or open fractures in our study. Comparison of spica casting to other treatments was confounded by differences in group characteristics, such as average age, high-energy/low-energy fracture, type of fracture, side, etc. Application of PSM created two equal and comparable treatment groups, and revealed a 10.5% higher rate of LLD in the spica cast treatment group (*P* = .05). Notably, 15 out of 16 cases that remained in the control group after PSM were children treated with TEN, the principal accepted alternative to spica casting for that age in our institution. The average age in those groups was around 4 years (Table 4), suggesting that cutoff as being the age for changing from spica treatment to TEN.

## Discussion

Our experience is that spica casting is an effective and reliable method for treating femoral shaft fractures in children. It had a union rate of 100%, with a 92.7% rate of normal leg length and an 85.4% complication-free rate. Only one of the 151 patients we treated with spica casting required subsequent surgery. Despite the good outcome achieved with spica casting compared to the other treatment options we examined, especially those appropriate for children above 4 years of age, spica casting had more side effects (mainly contact dermatitis) and more complications (mainly more cases of re-manipulation due to loss of reduction). It also had 10.5% more LLDs (>2 cm shortening or <1 cm lengthening). Nevertheless, we believe that these differences are still not convincing enough to rule out the use of spica casting, even in children above the age of 4 years, although they should be borne in mind and explained to the parents. The advantages of conservative treatment, such as the avoidance of general anesthesia on two occasions and the obviating of surgery, altogether make a compelling argument in its favor. On the other hand, the use of spica casting includes some disadvantages, such as a long period of immobilization, untoward side effects, the need for re-manipulation, and a higher risk for LLD.

A child whose femoral fracture is treated with the TEN technique achieves recovery milestones significantly faster than a child treated with traction and a spica cast, and the complication rate associated with nailing compares favorably with that associated with traction and application of a spica cast [18].

The TEN approach has some disadvantages that should be noted. One of them is that the child is expected to undergo two separate operations under general anesthesia. Our TEN group was too small to arrive at any firm conclusions, and our sole significant side effect of the TEN treatments was soft tissue irritation at the insertion site in one patient. The literature, however, more fully describes complications associated with TEN, such as re-fractures, delayed unions, varus or valgus malalignments, malrotation, nail tip irritations, broken interlocking screws, and proximal nail migration, reaching an overall complication rate of 11.7% [19-22].

Our experience with external fixation shows it to be an appropriate modality for pediatric femoral fractures, especially when dealing with an open fracture and a multitrauma-injured child. There is no need to convert to internal fixation. Surgical treatment for these fractures using various fixation devices (flexible nails, plating, or antegrade trochanteric nail) yielded highly satisfactory results with few complications in children who were 8 years and older, similar to others [23,24].

## Conclusion

In conclusion, the results of this work showed that good outcomes of pediatric femoral shaft fractures can be achieved by various methods of treatment and that the decision of which to use should be tailored to the needs of the specific child. We found that the TEN approach was superior to spica casting for children who had reached an average age of 4 years.

## Appendix

### The PSM method

The method involves two steps. In the first step, it estimates the chance of receiving each type of treatment for each patient in both groups (spica cast and others). This is done by running a logistic regression in which the dependent variable is treatment by spica casting or not (a binary variable). Its independent variables are the same as the explanatory variables of the second step of the method (e.g., age and gender). It then gives each patient a predicted probability of being treated by a spica cast (a ‘score’). The final part of the first step is to match between patients who have a similar (more or less) probability of being treated (in that step, an observation which has an exception probability of being treated will be excluded from the second step). The second step of the method is running the main regression, but only with patients among whom each patient in the control group has a predicted probability of being treated that is similar to a patient in the treatment group.

## Competing interests

The authors declare that they have no competing interests.

## Authors’ contributions

YS and OH contributed equally and each should be considered as first author. YS, OH, NS, AS, and UG carried out the conception, design, analysis, and interpretation of data; drafted the article; revised it critically for important intellectual content; and gave final approval of the version to be published. All authors read and approved the final manuscript.
